# EXPLORING SEX DIFFERENCES IN COGNITION IN OLDER BLACKS

**DOI:** 10.1093/geroni/igz038.1999

**Published:** 2019-11-08

**Authors:** DeAnnah R Byrd, Roland J Thorpe, Keith E Whitfield

**Affiliations:** 1 Wayne State University, Detroit, Michigan, United States; 2 Johns Hopkins, Baltimore, Maryland, United States

## Abstract

Previous literature suggest that women experience more dementia than men. However, it is unclear what accounts for these differences and whether sex differences exist among Blacks over time. We hypothesize that Black women will have worse cognitive outcomes than men and smoking may potentially explain these differences. Longitudinal data from the Baltimore Study of Black Aging-Patterns of Cognitive Aging was used to assess cognitive change over 33 months in five domains. The sample consisted of 602 community-dwelling Blacks, aged 48-92 years at baseline and 450 at follow-up. Findings indicated that Black women reported better vocabulary, working and verbal memory than Black men, controlling for age, education, smoking, and health status. These findings suggest that Black women may have some cognitive advantages in mid to later life compared to Black men. Future research should continue exploring longitudinal sex differences in cognitive domains among Blacks and the underlying drivers of these differences.

